# Gut-binding peptides as potential tools to reduce virus binding to honey bee gut surface proteins

**DOI:** 10.1128/aem.02418-24

**Published:** 2025-02-28

**Authors:** Ya Guo, Lincoln N. Taylor, Ruchir Mishra, Adam G. Dolezal, Bryony C. Bonning

**Affiliations:** 1 Department of Entomology and Nematology, University of Florida 166763 , Gainesville, Florida, USA; 2 Department of Entomology, University of Illinois at Urbana-Champaign, Champaign, Illinois, USA; UMR Processus Infectieux en Milieu Insulaire Tropical, Ste. Clotilde, France

**Keywords:** honey bee, *Apis mellifera*, gut-binding peptide, virus, *Israeli acute paralysis virus*, *Deformed wing virus*, dicistrovirus ORFx, *Dicistroviridae*, * Iflaviridae*

## Abstract

Colonies of the western honey bee, *Apis mellifera,* are severely impacted by a wide range of stressors, with *Varroa* mites and associated viruses being among the most serious threats to honey bee health. Viral load plays an important role in colony demise, with the iflavirus *Deformed wing virus* (DWV) and the dicistrovirus *Israeli acute paralysis virus* (IAPV) being of particular concern. By feeding adult honey bees on a phage display library to identify gut-binding peptides (R. Mishra, Y. Guo, P. Kumar, P. E. Cantón, C. S. Tavares, R. Banerjee, S. Kuwar, and B. C. Bonning, Curr Res Insect Sci, 1:100012, https://doi.org/10.1016/j.cris.2021.100012), we identified Bee midgut-Binding Peptide (BBP2.1), which shares 75% and 85% identity with regions on the DWV capsid protein and IAPV ORFx protein, respectively. These viral protein domains are likely to be instrumental in virus interaction with the honey bee gut. Pull-down assays with honey bee gut brush border membrane vesicles were used to confirm peptide-mCherry binding to the gut for BBP2.1 and the two similar virus-derived sequences, peptides BBP2.1^DWV^ and BBP2.1^IAPV^. *In vitro* competition assays showed that all three peptides compete with both IAPV and DWV virions for binding to honey bee gut-derived brush border membrane vesicles, suggesting that the three peptides and the two viruses bind to the same proteins. Ingestion of BBP2.1 reduced the movement of DWV, but not IAPV from the honey bee gut into the body and did not rescue IAPV-associated mortality. These results are discussed in relation to the biological function of IAPV ORFx and the potential utility of virus-blocking peptides for suppression of virus infection to reduce virus load and virus-associated honey bee mortality.

**IMPORTANCE:**

Each year, approximately 40% of managed honey bee hives in the United States are lost due to a variety of environmental stressors. Although increases in virus infection are among the most important factors resulting in colony loss, there are currently no effective tools for the management of virus infection in honey bees. In this study, we identified a peptide that binds to the gut of the honey bee and competes with two of the most important honey bee viruses, Israeli acute paralysis virus of bees (IAPV) and Deformed wing virus (DWV), for binding to gut proteins. *In vivo* competition between this peptide and DWV demonstrates the potential utility of gut-binding peptides for the protection of honey bees from virus infection for reduced virus-associated honey bee mortality.

## INTRODUCTION

The European honey bee (*Apis mellifera*) provides critical pollination services that are valued at $16 billion annually in the United States (1). Extensive colony losses have been attributed to multiple stress factors, particularly the Varroa mite (2) and associated bee viruses (3). More than 22 viruses are known to infect honey bees (4, 5), most of which have positive sense, single-strand RNA genomes, and belong to the order *Picornavirales*. These viruses include members of the family *Iflaviridae*, such as Deformed wing virus (DWV), sacbrood virus, Kakugo virus, Deformed wing virus-B (formerly Varroa destructor virus-1), and of the family *Dicistroviridae*, such as Israeli acute paralysis virus (IAPV), black queen cell virus (BQCV), Kashmir bee virus (KBV), and acute bee paralysis virus (ABPV). IAPV and DWV are both widespread and are transmitted by Varroa mites, via food and vertically from queen to larvae (6–9). DWV infections are dominant in Varroa epidemic areas (10) and have deleterious effects on honey bees at high viral loads (11). Both IAPV and DWV have been associated with colony losses (12, 13).

The viruses DWV and IAPV both have positive sense, single-strand RNA genomes, and nonenveloped icosahedral virions. The DWV genome encodes a single polyprotein from which four structural proteins (VP1-4) are generated. The C-terminal region of VP3 is folded to form a globular protruding (P) domain, which is exposed on the outside of the virion and is predicted to be involved in binding to the honey bee gut epithelium during virus entry (14). The IAPV genome contains two open reading frames (ORFs) that separately encode nonstructural and structural polyproteins (VP1-4). The two ORFs are separated by an intergenic region, and each ORF is translated via distinct internal ribosome entry site (IRES) elements (15). The VP1-3 proteins form the main virion capsid, with the smaller VP4 attached to the inner surface of the capsid (16).

Some dicistroviruses that have type-6b IRES elements translate two distinct proteins from overlapping 0 and +1 reading frames in the C-terminal ORF that encodes the structural proteins (17–19). In addition to the polyprotein comprised of the four structural proteins translated from the 0 reading frame, a protein called ORFx is produced from the +1 reading frame. Dicistroviruses that encode ORFx include viruses of the honey bee (IAPV, BQCV, KBV, and ABPV) along with Cricket paralysis virus (CrPV). Indeed, there has been strong selection pressure for the maintenance of ORFx in the honey bee viruses with 61 of the 92–94 amino acid ORFx proteins completely conserved in KBV, ABPV, and IAPV (17). While the discovery of ORFx triggered comprehensive analyses of the varied RNA-ribosome interactions that mediate reading frame selection (20, 21), the biological function of dicistrovirus ORFx remains to be established.

Brush border membrane vesicles (BBMV) are vesicles produced by homogenization and differential centrifugation from epithelial cells that have a brush border (22). As BBMV are enriched in proteins found on the surface of the epithelium, insect gut BBMV are commonly used for the identification of receptor proteins for pathogens that infect or are transmitted by insects (23).

Phage display libraries fed to insects have been successfully used to identify gut-binding peptides in a wide range of insects including honey bees (24). Following feeding on the phage library, guts are dissected and washed, and bound phage is then eluted and amplified for a second round of feeding and phage enrichment. The sequences of peptides displayed by eluted phage from each round of enrichment are determined via sequencing, and peptides of interest are identified by bioinformatics analysis as described previously (24). These gut-binding peptides can be used to impede pathogen-insect interactions. For example, the peptide SM1, derived from a filamentous f88.4 phage library, effectively inhibited entry of *Plasmodium berghei* into the *Anopheles stephensi* gut and salivary glands (25). Gut-binding peptides identified for the pea aphid (*Acyrthosiphon pisum*; Hemiptera) competed with the plant virus, *Pea enation mosaic virus* (Luteoviridae), for entry into the pea aphid vector (26). Similarly, gut-binding peptides identified in tobacco budworm (*Heliothis virescens*; Lepidoptera) impeded baculovirus infection (27).

The goal of the work described here was to identify honey bee gut-binding peptides that compete with IAPV and DWV for gut binding, as a foundation for their potential use in protecting honey bees from oral infection by these viruses. Having identified honey bee gut-binding peptides by *in vivo* screening of a Ph.D.-C7C phage display library as reported previously (24), we identified peptides with amino acid sequence similarity to IAPV and DWV proteins and evaluated the impact of selected peptides on virus infection. The ability of these peptides to compete with IAPV and DWV in pull-down assays was assessed, and impacts on viral load and honey bee survival were determined. The results from this study are discussed relative to virus-honey bee gut epithelium molecular interactions and the potential use of gut-binding peptides as a novel strategy to protect honey bees against viral infection.

## RESULTS

### Analysis of honey bee gut-binding peptides identified by NGS

**Fig 1:**
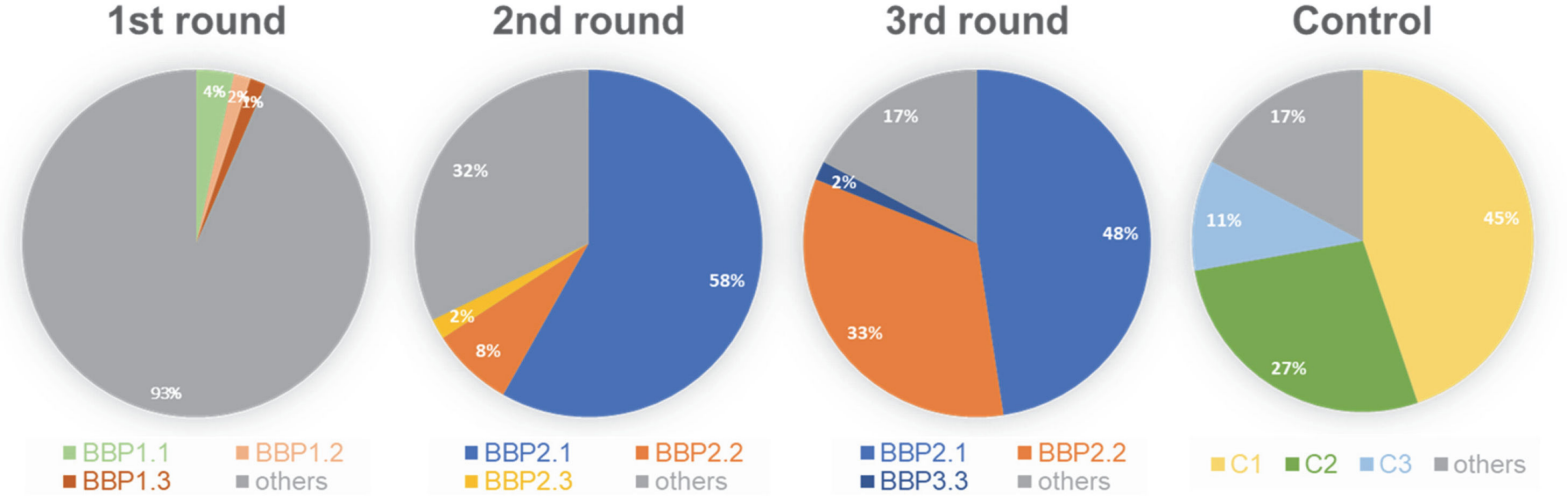
Enrichment of phage displaying honey bee gut-binding peptides including BPP2.1 during successive rounds of phage selection. Notable enrichment of BBP2.1 in blue and BBP2.2 in orange was observed after the second and third rounds of enrichment. Control represents the original, naïve phage display library.

Three rounds of phage enrichment were conducted for the isolation of honey bee gut-binding peptides. Next-generation sequencing resulted in ~4 million peptide sequences from eluted phages from each round of phage selection as reported previously (24). Of the top 40 most enriched phage-displayed peptides from each round (Fig. S1), two (BBP2.1 and BBP2.2) were significantly enriched from <1% in the first round to ~48% and 33% in the third round (Fig. 1; Table S1). Several highly abundant peptides following the first round of selection became less abundant as the screen progressed (Fig. 1).

### Several enriched peptides shared high similarity to viral capsid protein sequences

**Fig 2:**
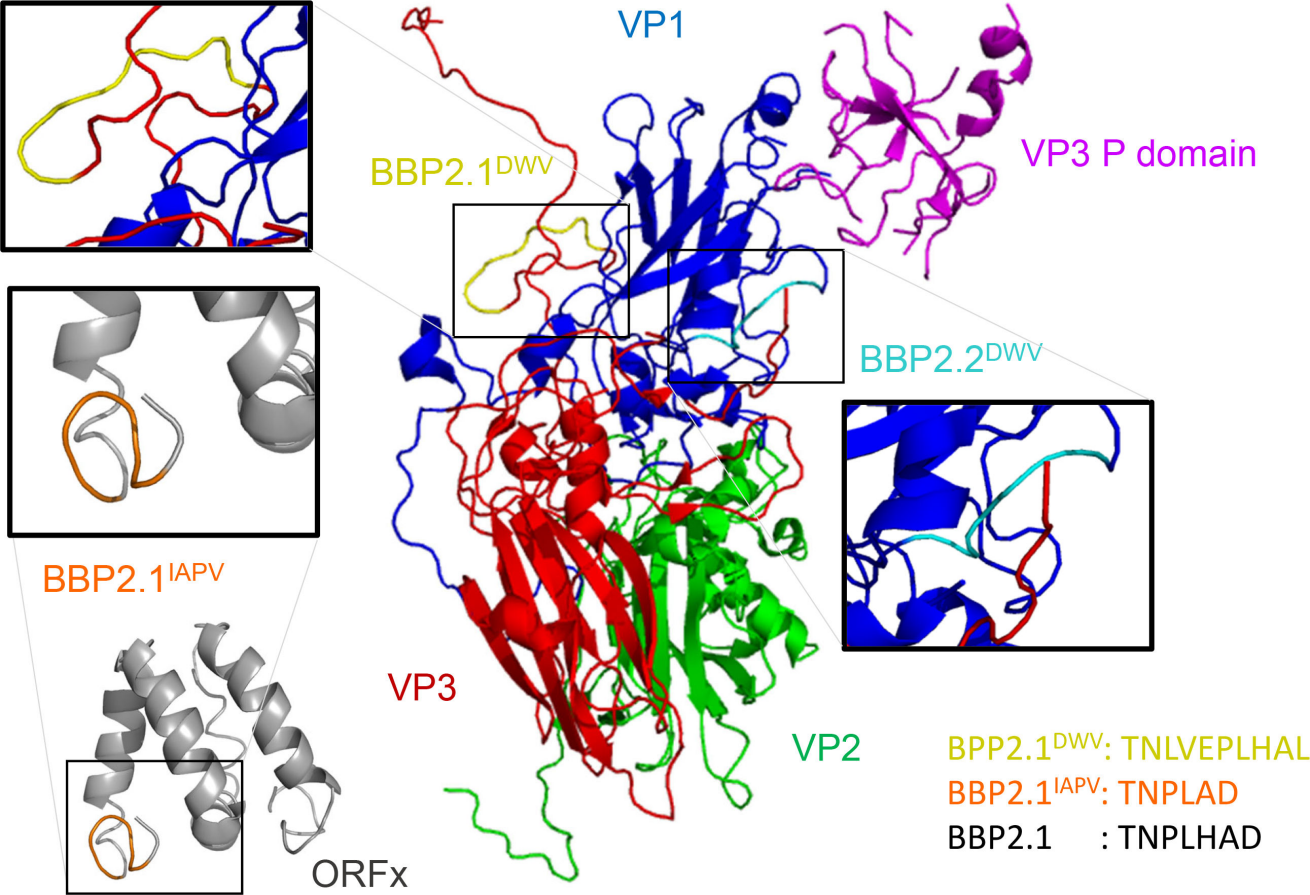
Similarity between honey bee gut-binding peptides and IAPV and DWV virion proteins. The positions of sequences with similarity to BBP2.1^DWV^ (yellow) in DWV VP3 and BBP2.2^DWV^ (pale blue) in DWV VP1 are indicated. The position of BBP2.1^IAPV^ (orange) in the IAPV ORFx protein is also indicated. The site of interaction of ORFx with the virion, if applicable, is unknown. The amino acid sequences of the three peptides BBP2.1^DWV^, BBP2.1^IAPV^, and the original peptide isolated from the phage display library, BBP2.1, are provided. The 3D structures of DWV structural proteins were obtained from the protein data bank (PDB) under PDB ID codes 5MUP (14). The structure of IAPV ORFx was predicted using ITASSER (28).

BLASTp was used to identify peptides similar to viral proteins, using the top 20 peptides from each round of enrichment that were predicted to be stable and hydrophilic (Table S2). The most enriched peptide, BBP2.1, shared 67% (6/9 aa) identity with a region of DWV VP3 and 86% (6/7 aa) identity with a region of the IAPV ORFx protein (Table 1; Fig. 2). Similarly, BBP2.2 shared 80% (4/5 aa) identity with a region of DWV VP1 (Table 1). The regions in DWV VP3 similar to BBP2.1^DWV^ and BBP2.2^DWV^ were also mapped to the DWV capsid protein structure (Fig. 2).

**TABLE 1:** Amino acid identities between the most enriched peptides and viral capsid sequences

BBP2.1	TN - - -PLHAD	BBP2.1	TNPLHAD	BBP2.2	FTLDGGS
	* * * * * *		* * * * * *		* * * *
**DWV VP3** **(BBP2.1 ^DWV^**)	TNLVEPLHAL	**IAPV ORFx** **(BBP2.1^IAPV^**)	TNPL -AD	**DWV VP1** **(BBP2.2 ^DWV^**)	LALD IGS

The ORFx protein of IAPV has two alpha helices with a 17 amino acid linker between them with 11 amino acid N- and 12 amino acid C-terminal extensions (Fig. 2). BBP2.1 maps to the C terminus of ORFx. All peptides mapped to flexible loop regions of the viral proteins (Fig. 2).

Given the similarity to honey bee gut-binding peptide sequences, these regions are likely to be instrumental in viral interaction with the surface of the honey bee gut. Based on the high similarity between BBP2.1 and the structural proteins of both DWV and IAPV, the properties of the three peptides BBP2.1, BBP2.1^DWV^, and BBP2.1^IAPV^ (Fig. 2) were determined. The amino acid sequences of BBP2.1 (7 amino acids; pI 5.05; Expasy), BBP2.1^IAPV^ (6 amino acids; pI 3.8), and BBP2.1^DWV^ (10 amino acids; pI 5.21) are highly similar (Table 1). BBP2.1 and BBP2.1^IAPV^ both have a high antigenic index (Fig. S2). BBP2.1 and BBP2.1^IAPV^ are hydrophilic, whereas BBP2.1^DWV^ has a more neutral hydrophilicity profile. The peptides also vary in the location of amphipathic regions (Fig. S2).

### *In vitro* confirmation of peptide binding to honey bee gut proteins

**Fig 3:**
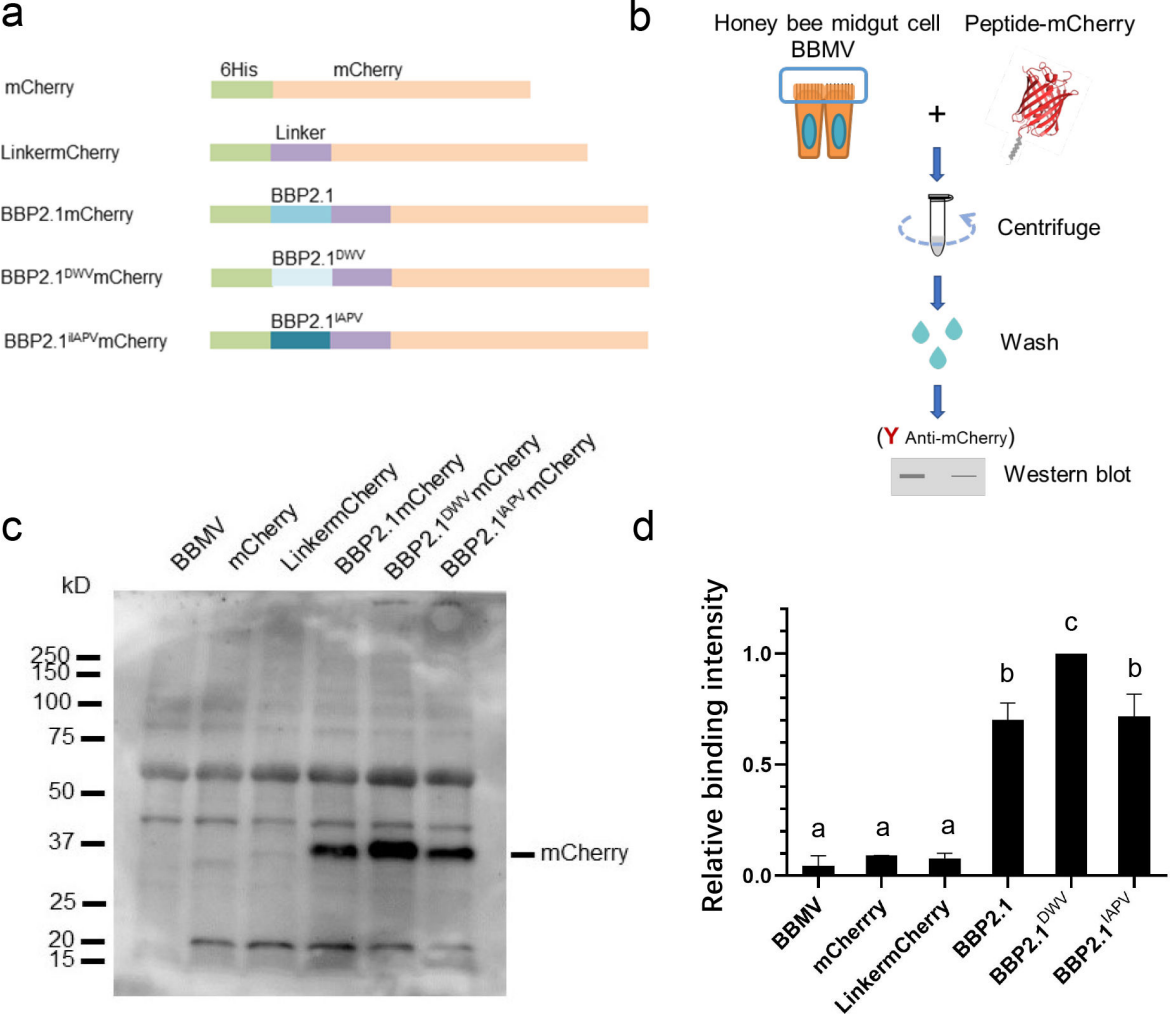
*In vitro* confirmation of BBP2.1-mCherry binding. (**a**) Constructs for mCherry, linker-mCherry, and peptide-mCherry are shown; 6His, histidine tag; linker, (AP)5. (**b**) Workflow for pulldown assays to assess the binding of peptide-mCherry and control proteins to BBMV. (c) In contrast to the negative controls (mCherry and linker-mCherry), pull down of the three peptide-mCherry fusions with honey bee gut-derived BBMV was confirmed by western blot with mCherry antibody. Molecular mass markers (kD) are indicated. (**d**) The relative intensity of mCherry bands from three replicate western blots (± SD) as determined by ImageJ (arbitrary units) is shown at right. Statistical differences were determined by ANOVA and Tukey’s multiple comparisons test. Different letters indicate significant differences among groups at *P* < 0.05, according to Tukey’s multiple comparisons test.

To confirm binding of the three BBP2.1 peptides (BBP2.1, BBP2.1^DWV^, and BBP2.1^IAPV^) to honey bee gut proteins, *in vitro* pull-down assays were conducted with honey bee gut-derived BBMV, which are enriched in gut surface proteins, and peptide-linker mCherry fusion proteins (Fig. 3a). For this experiment, purified peptide-mCherry and control proteins (Fig. S3) were incubated with BBMV, and bound fusion proteins were pelleted with BBMV by centrifugation. Proteins in the pellet were then examined by western blot for the presence of mCherry as an indication of peptide-mCherry binding (Fig. 3b). All three peptide-mCherry fusion proteins were pulled down with BBMV in these experiments (Fig. 3c and d). In contrast, mCherry and linker-mCherry were detected at very low levels in the pelleted BBMV. These results confirm that BBP2.1, BBP2.1^DWV^, and BBP2.1^IAPV^ bind to honey bee gut proteins under *in vitro* conditions.

### BBP2.1 peptides compete with honey bee virions for binding to honey bee BBMV

**Fig 4:**
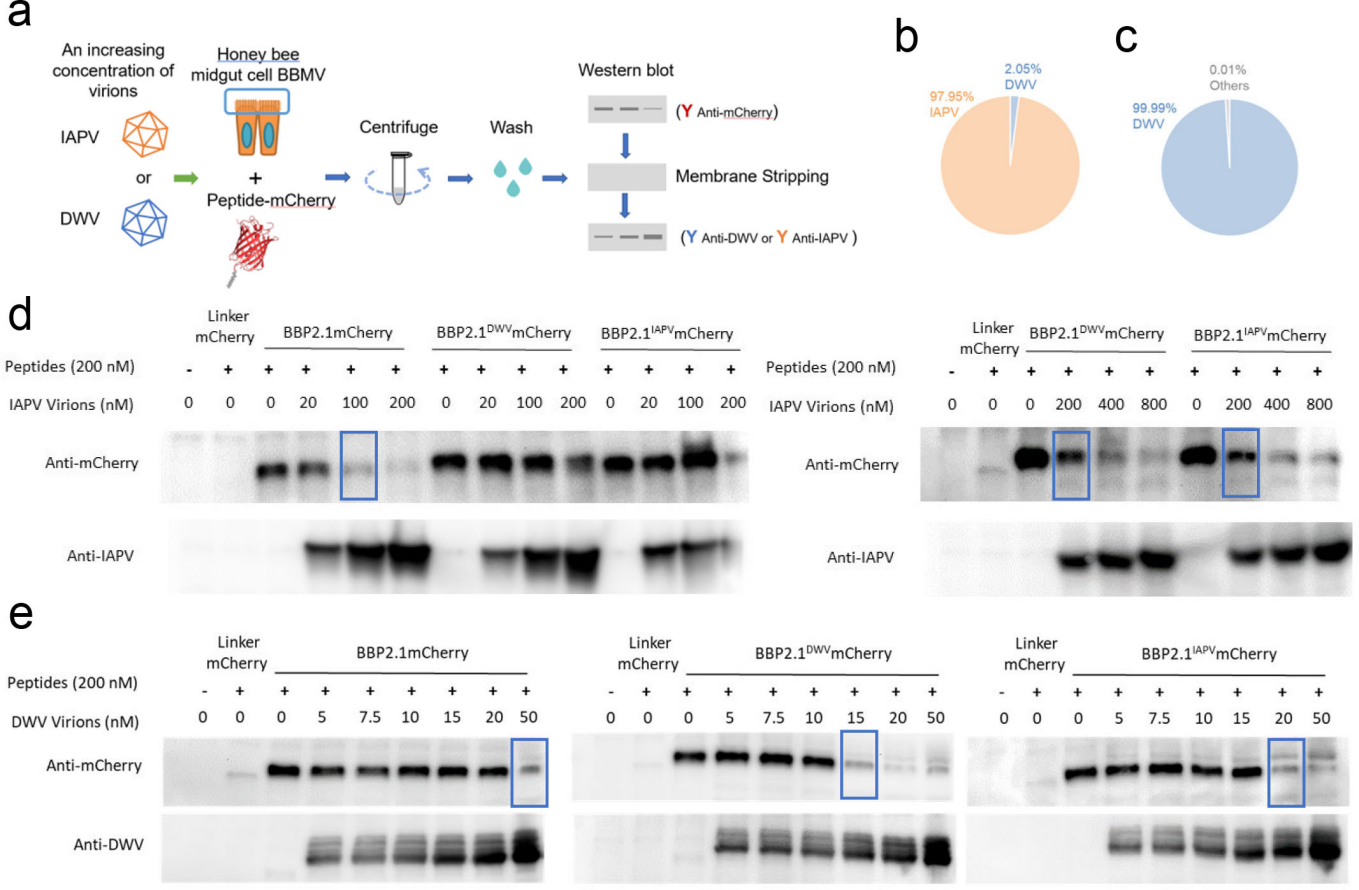
Competition between BBP2.1 peptides with virus for binding to honey bee gut-derived BBMV. (a) Workflow for competition pull-down assays. Pull-down assays with increasing amounts of IAPV or DWV virions were used. Pie charts representing the composition of (b) IAPV and (c) DWV stocks used for these assays. Pull-down assays with (d) IAPV and (e) DWV virions. Bands of decreased intensity indicative of peptide competition for virus binding to BBMV are boxed. Pull-down assays with linker-mCherry were used as a negative control, and the increasing amount of virus was visualized by western blot.

We assessed the ability of the peptides BBP2.1, BBP2.1^DWV^, and BBP2.1^IAPV^ to compete with IAPV or DWV virions for binding to the surface of the honey bee gut epithelium using BBMV. Virus stocks comprised of 97.95% IAPV virions and 99.99% DWV virions were used for these assays (Fig. 4). BBMVs were incubated with BBP2.1-mCherry fusion proteins with increasing concentrations of IAPV or DWV virions. Peptide-mCherry binding to BBMV in the presence of increasing concentrations of virions was evaluated by western blot with anti-mCherry antibody. All three peptides interfered with IAPV binding at a virion concentration range of 100–200 nM based on decreased band intensities (Fig. 4). BBP2.1^IAPV^ and BBP2.1^DWV^ bound to BBMV more strongly than BBP2.1 in this *in vitro* assay, as higher concentrations of IAPV virions were required to compete with BBP2.1^IAPV^ and BBP2.1^DWV^. The three peptide-mCherry fusions also competed with DWV virions for BBMV binding within a narrow range of virion concentrations, 15–50 nM (equivalent to ~9 × 10^9^ to 3 × 10^10^ virions). These results suggest that the three peptides and the two viruses bind to and compete for the same honey bee gut surface proteins.

### BBP2.1 interferes with DWV virus movement from the gut into the hemocoel

As the three BBP2.1 peptides compete with virions *in vitro* for binding to bee gut-derived BBMV, we explored whether BBP2.1 interferes with DWV movement from the honey bee gut into the hemocoel. Because DWV is widespread in honey bees, we fed an increasing amount of DWV virions to determine the minimum amount of added virus needed to be able to distinguish inoculated virus from background DWV infections. Feeding of 1 nM of DWV virions in a volume of 15 µL was sufficient for the detection of significantly higher DWV viral load in the gut, and the body minus the gut (Fig. S4).

To evaluate the ability of BBP2.1 to compete with virions for gut binding, we fed individual bees on 1 nM DWV virions with or without BBP2.1. Infections were allowed to continue for a period of 16 h, and the whole body without gut was used for the determination of virus genome equivalents by RT-qPCR. We thus quantified the amount of virus that had spread beyond the gut, by removing any ingested virions retained in the gut lumen along with any virions present in the gut epithelium.

**Fig 5:**
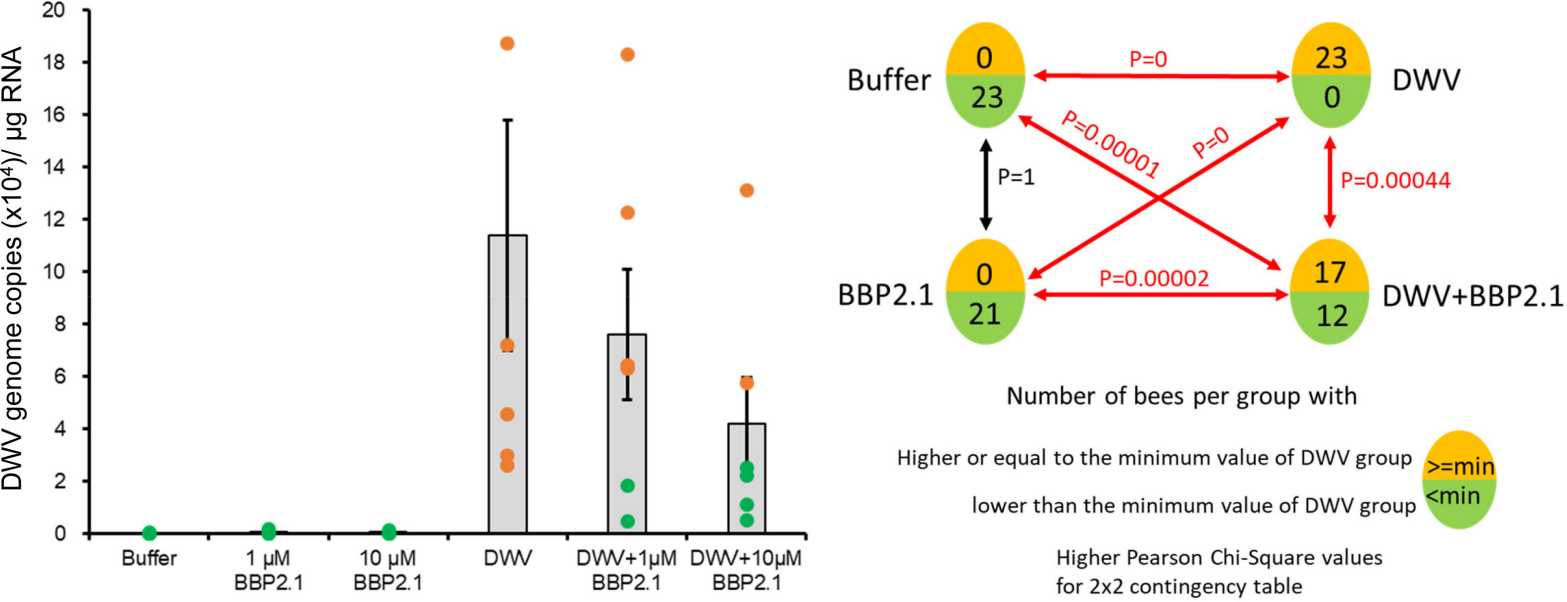
BBP2.1 competes with DWV for *in vivo* binding to the honey bee gut. The DWV load in the honey bee minus gut was determined for bees in each treatment. The lowest virus load in the DWV-only treatment was used as the threshold to distinguish between low and high virus loads. The total number of bees in each treatment above and below this threshold was determined. The mean and SE of one experiment are shown with DWV loads below the threshold indicated in green, and above the threshold in orange. Five independent experiments were conducted using three to six honey bees per treatment from different colonies with different levels of background DWV infection. Pearson χ test *P* values are shown at right for the 2 × 2 contingency table.

Feeding honey bees on a mixture of DWV virions with 0, 1, or 10 µM BBP2.1 (representing a ratio of 1,000 peptides per virion at 1 µM and 10,000 per virion at 10 µM) resulted in a trend of decreasing DWV load in the bee body compared with the DWV alone treatment (Fig. 5). For honey bees with virus loads below the threshold (i.e., below the minimum viral load in the DWV-only treatment), BBP2.1 was considered to have interfered with virus infection. This approach was taken for analysis to ensure that background DWV infection of the bees did not confound the analysis of the results. Feeding DWV with BBP2.1 significantly reduced the number of bees with a high DWV infection (*P* = 0.00044; Pearson χ^2^ test, 2 × 2 contingency table; Fig. 5).

### BBP2.1 did not reduce IAPV-induced honey bee mortality

**Fig 6:**
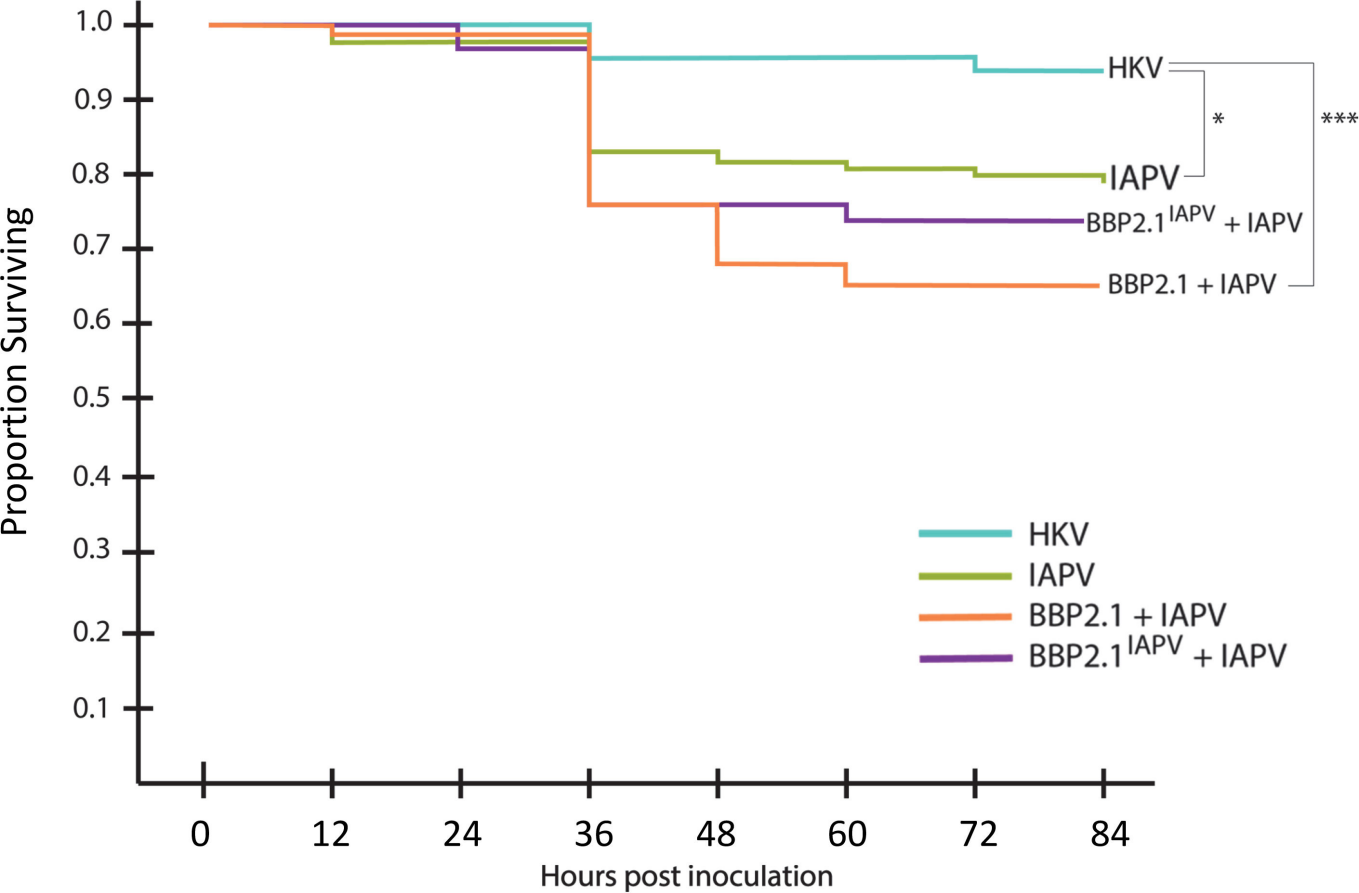
BBP2.1 and BBP2.1^IAPV^ had no impact on honey bee survival when fed to bees with IAPV. Survival was significantly reduced by IAPV and by BBP2.1+IAPV relative to heat-killed virus (HKV; *P* = 0.016* and *P* = 0.00049***, respectively; pairwise Cox proportional hazard and Benjamini-Hochberg correction). A total of 120 bees per treatment were used for this experiment.

Following oral inoculation of individual bees, no differences were observed in the survival of honey bees fed peptide (BBP2.1 or BBP2.1^IAPV^) plus IAPV compared with bees fed IAPV alone (Fig. 6). However, survival at 84 h for the IAPV treatment was higher than expected at 80%. There were also no significant differences in viral load between any of the treatments for surviving bees sampled at 36 h (*P* = 0.63; one-way ANOVA) (Fig. S5).

## DISCUSSION

The motivation for this work was to identify honey bee gut-binding peptides that compete with IAPV and DWV for binding to the surface of the honey bee gut epithelium on the basis that such peptides have potential use for the management of honey bee viral disease. Bioinformatics analysis of gut-binding peptides enriched during three rounds of phage library screening resulted in the identification of several peptides with high identity to viral structural protein sequences. As the most highly enriched peptide BBP2.1 (TNPLHAD) has amino acid sequence similarity to specific domains in both DWV VP3 and IAPV ORFx, we focused on this peptide and the IAPV and DWV sequence variants for the current study. Although competition between the three BBP2.1 peptides (BBP2.1 from the phage library screen, BBP2.1^IAPV^ comprised of the similar IAPV sequence, and BBP2.1^DWV^, the DWV sequence) with IAPV and DWV was shown in pull-down assays with BBMV under *in vitro* conditions, significant competition was only seen under *in vivo* conditions for BBP2.1 and DWV.

### IAPV ORFx

Although the biological function of the dicistrovirus ORFx is unknown, the high degree of identity between BBP2.1 and ORFx may provide insights. Viral infectious clones provide valuable tools for the assessment of gene function and an infectious clone of the dicistrovirus CrPV has been constructed (29). Although CrPV ORFx has no appreciable homology to other dicistrovirus ORFx proteins, it shares the C-terminal, alpha-helical segment, which is predicted to be an ORFx transmembrane domain (18). The replication of mutants of an infectious clone deficient in ORFx was indistinguishable from the unmodified infectious clone in cell culture, indicating that ORFx is not required for virus replication. The ORFx protein is associated with ER and Golgi membranes in S2 cells (29), demonstrating the capacity of the alpha-helical domain of CrPV ORFx to insert into membranes. Expression of ORFx was confirmed in honey bee pupae 6 and 24 h after injection with IAPV virions (21). The detection of two ORFx-derived peptides (common to both IAPV and KBV) required use of the highly sensitive and specific, multiple reaction-monitoring MS, suggesting either that the protein is unstable or that ORFx is expressed at a very specific stage during virus replication. The ORFx of *Solenopsis invicta virus 1* was expressed when fused to maltose-binding protein (MBP) (30), with the MBP stabilizing ORFx.

The translation of both IAPV ORF2 and ORFx is driven by the IGR IRES (18) with the ORFx protein and ORF2 polyprotein produced only when virion packaging is required (15). This suggests that ORFx may function during packaging or downstream during infection. Our data suggest that ORFx functions during infection of the honey bee gut epithelium with the C-terminus binding to the microvillar membrane. Whether ORFx inserts into the membrane remains to be determined.

### *In vivo* virus-peptide competition

DWV infection of honey bees is ubiquitous, which presents a major challenge for the assessment of the ability of gut-binding peptides to compete with virus under *in vivo* conditions. The DWV infection levels can be highly variable, and the impact of such background infections on the ability of inoculated virus to replicate is unknown. In contrast, infection with IAPV, which results in honey bee mortality, provides a useful phenotypic measure to assess the impacts of gut-binding peptides on virus infection. However, BBP2.1 and BPP2.1^IAPV^ failed to rescue IAPV infection in our experiments.

Clearly, the *in vivo* experiments with IAPV did not reflect the *in vitro* competition demonstrated between IAPV virions and peptides for binding to honey bee gut-derived BBMV. This suggests that although virus and peptide compete for binding, additional *in vivo* events override the competition effect. The *in vivo* experiments showed a trend, suggesting that BBP2.1 and BPP2.1^IAPV^ might increase, rather than decrease IAPV-induced mortality. This result, along with the similarity of the peptides to IAPV ORFx, may shed light on the function of the protein encoded by ORFx: BBP2.1 is similar to the C terminal region of the ORFx protein, indicative of a role of this protein in binding to the honey bee gut. If the ORFx protein also attaches to the surface of the IAPV virion, it could serve to provide an additional anchor to facilitate gut binding by the virus. Alternatively, if peptide binding to the gut surface results in receptor-mediated endocytosis, virus uptake into the gut epithelial cells could be increased. It is possible that peptides with similarity to viral structural proteins could facilitate rather than impede gut binding. It is also possible that insufficient peptide was delivered to the gut surface, following oral delivery for competition with IAPV virions to be observed.

### Receptor and receptor-binding domains

Both DWV and IAPV are hypothesized to invade the midgut epithelial cells of honey bees via receptor-mediated endocytosis, but the proteins that mediate virus entry and the molecular mechanisms involved have yet to be elucidated. Gut-binding peptides with similarity to pathogen surface proteins can be used in UV-crosslinking assays to determine candidate pathogen receptors. The identification of honey bee gut-binding peptides with similarity to viral structural protein sequences highlights the likely importance of these domains on VP1 and VP3 for the association of DWV with the gut epithelium. We hypothesize that the regions of DWV VP3 and IAPV ORFx with similarity to BBP2.1 function in binding of the virus to the surface of the honey bee gut epithelium. Notably, sequences similar to that of BBP2.1^IAPV^ (TNPLAD) were also found in the ORFx of two other dicitrosviruses, namely acute bee paralysis virus (TNPSID) and Solenopsis invicta virus 1 (TNPLTQ), suggesting that these ORFx proteins bind a conserved gut surface protein in their respective bee and ant hosts. UV-crosslinking using peptide BBP2.1 and BBMV could be used to identify candidate gut-binding partners and receptors for these viruses, as described previously (31, 32). Five additional peptides enriched during the phage display screen mapped to the C-terminal *P* domain of VP3 that was previously predicted to be involved in virus binding, supporting a role for this domain in gut binding (14).

### Concluding remarks

In the present study, we demonstrated gut-binding peptide competition with DWV but not with IAPV under *in vivo* conditions. The identification of honey bee gut-binding peptides with similarity to DWV and IAPV structural proteins including ORFx provides valuable insight into viral domains likely to be involved with receptor binding. Given the alpha-helical transmembrane domain and C-terminal gut-binding sequence of ORFx, we hypothesize that this protein associates with viral structural proteins during virion assembly and functions to enhance viral uptake into gut epithelial cells by binding to the gut surface. The biological function of ORFx, confirmation of the putative virion binding domains, and identification of IAPV and DWV gut receptors all warrant further investigation.

A promising approach for the downstream application of peptides that compete with honey bee viruses is the use of symbiotic bacteria that reside in the honey bee gut for peptide delivery. Indeed, the utility of this paratransgenic approach has already been demonstrated for bacterial delivery of gene-silencing RNAs into the honey bee gut (33). We expect that continuous bacteria-mediated peptide delivery proximal to the surface of the gut epithelium would be ideal for *in vivo* competition between honey bee viruses and gut-binding peptides and could, in the long term, be employed for the protection of honey bee health.

## MATERIALS AND METHODS

### Insects

Honey bees, *A. mellifera* (Insecta: Hymenoptera: Apidae), were provided by the Honey Bee Research and Extension Lab at the University of Florida (Gainesville, FL, HBREL). Pupae and newly emerged bees were raised in a chamber at 32°C, 70% humidity.

### Identification of honey bee gut-binding peptides of interest

Peptide nucleotide sequences acquired from each round of phage enrichment were converted to amino acid sequences (aa), and unique seven aa sequences with counts extracted for further analysis. Normalization, calculation of enrichment, removal of false positives using the SAROTUP suite of tests, and prediction of stability and hydrophilicity using Peptides version 2.4.2 (R package) were as described previously (24) (see also the supplemental methods). BLASTp (34) was then conducted with the enriched peptides predicted to be hydrophilic and stable against IAPV and DWV structural protein sequences to identify peptides with aa sequence similarity to the viral structural proteins. The 3D structures of DWV structural proteins (14) were obtained from the PDB online database (PDB ID code 5MUP). The structure of the IAPV ORFx protein was predicted by the use of ITASSER software (https://zhanglab.ccmb.med.umich.edu/) (28). Regions in IAPV and DWV capsid proteins with similarity to honey bee gut-binding peptides were mapped and visualized using PyMOL software.

### Brush border membrane vesicle (BBMV) preparation

A total of 50 adult honey bee midguts were used for BBMV preparation as described previously (22). The use of BBMVs, which are highly enriched in gut surface proteins, facilitates the analysis of pathogen interactions with the insect gut surface (23). The presence of intracellular proteins that are also present in BBMV preparations should also be taken into account (35, 36). For BBMV preparation, midgut samples were transferred into a 20 mL Dounce homogenizer with 15 mL ice-cold MET buffer A (300 mM mannitol, 17 mM Tris HCl pH 7.5, 5 mM EGTA, 1/50 protease inhibitor, 1 mM). After homogenizing and centrifuging at 4°C, 1,000 g for 10 min, the pellet was removed. A 15 mL volume of 24 mM MgCl_2_ was added to the supernatant, and the mixture was centrifuged at 4°C, 2,500 g for 15 min. The supernatant was then transferred into a clean tube and centrifuged at 4°C, 30,000 × *g* for 30 min. The pellet was resuspended in 7.5 mL MET buffer A by homogenization, treated with MgCl_2_, and centrifuged at 2,500 × g for 15 min to further remove contaminants. Finally, the second (30,000 × *g*) pellet-containing BBMV was resuspended in 2 mL cold MET buffer A. Protein concentrations were determined by Bradford assay. As aminopeptidase N is among the most abundant proteins on the surface of the insect gut, the enrichment of gut surface proteins in the BBMV samples was evaluated by comparing the aminopeptidase activity in the BBMV samples with that of the gut homogenates using standard procedures (37).

### Production of peptide-mCherry fusions

Peptide-mCherry fusions were constructed in the pBAD/HisB vector (Invitrogen, San Diego, CA) with an N terminal His-tag. A honey bee gut-binding peptide (BBP2.1) and two virus-derived peptides (BBP2.1^DWV^ and BBP2.1^IAPV^) were fused at the N-terminus of mCherry and inserted into pBAD/HisB between the SacI and HindIII sites. The pmCherry construct (Clontech, Mountain View, CA) was used as a PCR template. A forward primer containing a SacI site, the peptide sequence, a linker sequence (AP)5 and the 5’ end of the mCherry sequence, and a reverse primer complementary to the 3’ end of the mCherry with a HindIII site were used to obtain different peptide-mCherry fusion genes, and a total of 5 different mCherry versions (mCherry, Linker-mCherry, BBP2.1-mCherry, BBP2.1^DWV^-mCherry, BBP2.1^IAPV^-mCherry) were constructed. Primer sequences used for vector generation are provided in Table S3. The PCR products were purified using a Wizard SV Gel and PCR Clean-Up kit (Promega, Madison, WI, USA), digested with SacI and HindIII restriction enzymes, cleaned using a QIAEX II Gel Extraction Kit (Qiagen, Germantown, MD, USA), and ligated to the linearized pBAD/HisB. All inserted fragments were sequenced to confirm no gene frameshift or other mutations. Top 10 *E. coli* competent cells (Thermo Fisher Scientific, Waltham, MA, USA) were used for the expression of fusion proteins. Protein purification was carried out using the Capturem His-Tagged Purification Maxiprep Kit (Clontech, Mountain View, CA, USA). Imidazole was filtered out from all protein samples using Amicon Ultra-0.5 mL centrifugal filters Ultracel-3K (Millipore Sigma, St. Louis, MO, USA).

### Production and purification of virus stocks

Amplification of honey bee viruses in pupae for the production of virus stocks was optimized as previously described (38). Pupae were injected at the white eye stage with 1 µL virus homogenate or RNA transcripts and incubated at 32°C for 5–7 days. One hundred injected pupae were homogenized in 60 mL PBS buffer. Cellular debris was removed by two rounds of centrifugation (15,000 × *g* for 5 min, 4°C). The supernatant was treated with 0.3 volumes of chloroform: isoamyl alcohol (24:1), and viral particles were enriched by 2.3% NaCl-7% PEG 8000 precipitation. The PEG-virion pellet was recovered by centrifugation at 4°C, 15,000 × *g* for 30 min, and the resulting pellet was re-suspended in 10 mL TBS buffer, and centrifuged twice (15,000 × *g* for 5 min, 4°C) to remove PEG. The supernatant was filtered through a 0.2 µm filter, and virus particles were concentrated to ~2 mL with a microcon cartridge 100Y (100 kDa cutoff; Amicon, Sigma Aldridge, St Louis, MO, USA) at 4,000 × *g* for 30 min. Finally, virions were cleaned by passing through a cushion of 20% sucrose/TES buffer, at 4°C, 100,000 × *g* for 4 h. Virus particles were diluted in PBS and stored at −80°C.

For the production of the DWV stock, a full-length infectious clone of DWV (39) allowed for the rapid production of numerous DWV virions in bee pupae. The infectious clone was used to produce RNA transcripts that were later injected into bee pupae (39). Virus titers in samples extracted from injected pupae were assessed by RT-qPCR as described below. The IAPV and DWV virion samples were used to evaluate the ability of bee gut-binding peptides (BBPs) to competitively inhibit the binding of the virus to the honey bee gut.

### RT-qPCR

For quantification of viral genome equivalents, total RNA was extracted from purified virus samples or individual bees according to the PureLink RNA Mini Kit protocol (Ambion; Thermo Fisher Scientific). After DNase treatment, 1 µg RNA was reverse transcribed into cDNA with random hexamers (Invitrogen SuperScript III First-Strand Synthesis System, Waltham, MA, USA). Viral genomic copies were quantified by absolute real-time PCR on a CFX96 qPCR System (Bio-Rad, Hercules, CA, USA). The PCR reaction was carried out in a 20 µL volume containing 5 µL SsoAdvanced Universal SYBR Green Supermix (Bio-Rad), 1.25 µM of each primer, 5 µL cDNA, and sterile water. Virus-specific PCR primers from previous reports were used for the detection of six honey bee viruses: DWV, IAPV, ABPV, BQCV, KBV, and SBV (38) (Table S4). Standard curves for each virus were generated separately by serial dilution of a universal standard reference (USR) plasmid that contains six relevant viral amplicon sequences (38).

### Peptide pull-down and virion competition assays

The ability of the three BBP2.1 peptides to bind to honey bee gut-derived BBMV was evaluated by *in vitro* pull-down assay with peptide-mCherry fusions, as previously described (31). BBMV protein (10 µg) and BBP2.1-mCherry test proteins (200 nM), mCherry, or linker-mCherry (negative controls) were incubated with BBMV in 100 µL binding buffer (PBS, 0.1% BSA, 0.1% Tween 20, pH 7.5) for 1.5 h. BBMV alone was used as an additional negative control. BBMV along with any bound proteins were then pelleted by centrifugation at 4°C, 21,000 × *g* for 20 min. The pellet was resuspended in 100 µL binding buffer and centrifuged to remove unbound proteins. This step was conducted three times. The final pellet was boiled in 10 µL SDS-sample buffer for 5 min, centrifuged briefly, and proteins separated in a 10% SDS-PAGE gel. The proteins were transferred to a PVDF membrane for western blot with anti-mCherry antibody (Novus Biologicals, Littleton, CO, 1:5000 dilution) and a secondary HRP conjugated anti-rabbit antibody (Thermo Scientific, dilution 1:5000). Blots were visualized by use of the SuperSignal West Femto Chemiluminescent kit (Thermo Fisher Scientific; Waltham, MA). The intensities of mCherry bands of the expected size were determined using ImageJ software (40). The pull-down assay was repeated three times with ANOVA, and Tukey’s multiple comparisons test was used for the analysis of statistical differences between band intensities.

For competition assays, a similar procedure was followed with 10 µg BBMV and 200 nM BBP2.1-mCherry peptides, but with increasing virion concentrations for IAPV (20 to 800 nM) or DWV (5 to 50 nM). Peptide and honey bee BBMV only served as negative control treatments. Following the detection of peptide-mCherry fusions, the membrane was stripped and re-probed with anti-IAPV antibody (38) or anti-DWV antibody (purified anti-DWV VP1 clone A IgG [39]).

### Assessment of peptide interference with DWV movement from the gut to the hemocoel

To determine the amount of virus required for oral inoculation of honey bees to be able to distinguish between virus fed to bees and background DWV infection levels, individual, newly emerged bees were fed with 15 µL TBS buffer containing 25% sucrose and 0.1–100 nM purified DWV virions. Treated bees were raised individually in isolation cages to avoid virus cross-contamination. After 16 h, the honey bee gut was removed, and the viral load in the remaining body was assessed by RT-qPCR.

Biotin-labeled BBP2.1 peptides were synthesized at Genemed Synthesis (San Antonio, TX, USA). Newly emerged bees were individually fed on 1 nM DWV virions (15 µL) with or without biotin-BBP2.1 at 0.1 and 10 µM in a volume of 15 µL. Virus only, buffer only, and peptide only were used as control treatments. Five independent experiments were conducted using three to six honey bees per treatment from different colonies with different wild-type background DWV levels to account for variable background levels of DWV in individual bees. For analysis of the resulting data, the lowest virus load in the DWV-only treatment was used as the threshold to distinguish between low and high virus loads. The number of bees in each treatment above and below this threshold was determined and a Pearson χ^2^ test was conducted for the resulting 2 × 2 contingency table.

### *In vivo* peptide and IAPV competition assays

Bees (1 day old) were hand fed on 10 µL of inoculum containing 100 µM of peptide (BPP2.1 or BPP2.1^IAPV^) with IAPV (2,100 genome equivalents in 10 µL, 1.5 nM; expected LC_50_ dose), IAPV alone, or heat-killed virus at the same concentration in 50% sucrose solution. Heat-killed virus was generated by incubating the virus at 95°C for 120 min. Bees were maintained in plastic cube cages, with 20 bees per cage and 60 bees per treatment. This experiment was repeated for a total of 120 bees per treatment. Survival was monitored every 12 h for 84 h, and dead bees were removed and frozen. A total of 18 live bees from each treatment were collected at 36 h post-inoculation and frozen to assess viral loads. From these bees, randomly selected subsets of eight bees per treatment were used to assess viral levels. Survival data were analyzed by pairwise Cox proportional hazards models and Benjamini-Hochberg correction. The viral loads for these bees were determined by RT-qPCR with reference to the universal standard (38) and compared using a one-way ANOVA.

## Data Availability

All data associated with the phage display screen in honey bees are provided as described in reference 24. Data sets for each round of phage enrichment can be found at the following site: https://doi.org/10.6084/m9.figshare.13667630.v1 All additional associated data are presented within the article and supplemental material.

## SUPPLEMENTAL MATERIAL

Supplemental material aem.02418-24-s0001.pdf Figures S1 to S5, Tables S1 to S4, and supplemental methods.

ASM does not own the copyrights to Supplemental Material that may be linked to, or accessed through, an article. The authors have granted ASM a non-exclusive, world-wide license to publish the Supplemental Material files. Please contact the corresponding author directly for reuse.

## References

[1] Calderone NW. 2012. Insect pollinated crops, insect pollinators and US agriculture: trend analysis of aggregate data for the period 1992-2009. PLoS ONE 7:e37235. doi:10.1371/journal.pone.0037235 22629374 PMC3358326

[2] Rosenkranz P, Aumeier P, Ziegelmann B. 2010. Biology and control of Varroa destructor. J Invertebr Pathol 103 Suppl 1:S96–119. doi:10.1016/j.jip.2009.07.016 19909970

[3] Galbraith DA, Yang X, Niño EL, Yi S, Grozinger C. 2015. Parallel epigenomic and transcriptomic responses to viral infection in honey bees (Apis mellifera). PLoS Pathog 11:e1004713. doi:10.1371/journal.ppat.1004713 25811620 PMC4374888

[4] Chen YP, Siede R. 2007. Honey bee viruses. Adv Virus Res 70:33–80. doi:10.1016/S0065-3527(07)70002-7 17765703

[5] Runckel C, Flenniken ML, Engel JC, Ruby JG, Ganem D, Andino R, DeRisi JL. 2011. Temporal analysis of the honey bee microbiome reveals four novel viruses and seasonal prevalence of known viruses, nosema, and crithidia. PLoS ONE 6:e20656. doi:10.1371/journal.pone.0020656 21687739 PMC3110205

[6] Chen YP, Pettis JS, Corona M, Chen WP, Li CJ, Spivak M, Visscher PK, DeGrandi-Hoffman G, Boncristiani H, Zhao Y, et al.. 2014. Israeli acute paralysis virus: epidemiology, pathogenesis and implications for honey bee health. PLoS Pathog 10:e1004261. doi:10.1371/journal.ppat.1004261 25079600 PMC4117608

[7] Mazzei M, Carrozza ML, Luisi E, Forzan M, Giusti M, Sagona S, Tolari F, Felicioli A. 2014. Infectivity of DWV associated to flower pollen: experimental evidence of a horizontal transmission route. PLoS ONE 9:e113448. doi:10.1371/journal.pone.0113448 25419704 PMC4242645

[8] Yue C, Schröder M, Gisder S, Genersch E. 2007. Vertical-transmission routes for deformed wing virus of honeybees (Apis mellifera). J Gen Virol 88:2329–2336. doi:10.1099/vir.0.83101-0 17622639

[9] Amiri E, Kryger P, Meixner MD, Strand MK, Tarpy DR, Rueppell O. 2018. Quantitative patterns of vertical transmission of deformed wing virus in honey bees. PLoS ONE 13:e0195283. doi:10.1371/journal.pone.0195283 29596509 PMC5875871

[10] Martin SJ, Highfield AC, Brettell L, Villalobos EM, Budge GE, Powell M, Nikaido S, Schroeder DC. 2012. Global honey bee viral landscape altered by a parasitic mite. Science 336:1304–1306. doi:10.1126/science.1220941 22679096

[11] de Miranda JR, Genersch E. 2010. Deformed wing virus. J Invertebr Pathol 103:S48–S61. doi:10.1016/j.jip.2009.06.012 19909976

[12] Chejanovsky N, Ophir R, Schwager MS, Slabezki Y, Grossman S, Cox-Foster D. 2014. Characterization of viral siRNA populations in honey bee colony collapse disorder. Virology (Auckl) 454–455:176–183. doi:10.1016/j.virol.2014.02.012 24725944

[13] Cox-Foster DL, Conlan S, Holmes EC, Palacios G, Evans JD, Moran NA, Quan P-L, Briese T, Hornig M, Geiser DM, Martinson V, vanEngelsdorp D, Kalkstein AL, Drysdale A, Hui J, Zhai J, Cui L, Hutchison SK, Simons JF, Egholm M, Pettis JS, Lipkin WI. 2007. A metagenomic survey of microbes in honey bee colony collapse disorder. Science 318:283–287. doi:10.1126/science.1146498 17823314

[14] Škubník K, Nováček J, Füzik T, Přidal A, Paxton RJ, Plevka P. 2017. Structure of deformed wing virus, a major honey bee pathogen. Proc Natl Acad Sci USA 114:3210–3215. doi:10.1073/pnas.1615695114 28270616 PMC5373406

[15] Bonning BC, Miller WA. 2010. Dicistroviruses. Annu Rev Entomol 55:129–150. doi:10.1146/annurev-ento-112408-085457 19961327

[16] Mullapudi E, Přidal A, Pálková L, de Miranda JR, Plevka P. 2016. Virion structure of Israeli acute bee paralysis virus. J Virol 90:8150–8159. doi:10.1128/JVI.00854-16 27384649 PMC5008081

[17] Firth AE, Wang QS, Jan E, Atkins JF. 2009. Bioinformatic evidence for a stem-loop structure 5’-adjacent to the IGR-IRES and for an overlapping gene in the bee paralysis dicistroviruses. Virol J 6:193. doi:10.1186/1743-422X-6-193 19895695 PMC2777877

[18] Sabath N, Price N, Graur D. 2009. A potentially novel overlapping gene in the genomes of Israeli acute paralysis virus and its relatives. Virol J 6:144. doi:10.1186/1743-422X-6-144 19761605 PMC2754452

[19] Wang QS, Jan E. 2014. Switch from cap- to factorless IRES-dependent 0 and +1 frame translation during cellular stress and dicistrovirus infection. PLoS One 9:e103601. doi:10.1371/journal.pone.0103601 25089704 PMC4121135

[20] Au HH, Cornilescu G, Mouzakis KD, Ren Q, Burke JE, Lee S, Butcher SE, Jan E. 2015. Global shape mimicry of tRNA within a viral internal ribosome entry site mediates translational reading frame selection. Proc Natl Acad Sci USA 112:E6446–55. doi:10.1073/pnas.1512088112 26554019 PMC4664324

[21] Ren Q, Wang QS, Firth AE, Chan MMY, Gouw JW, Guarna MM, Foster LJ, Atkins JF, Jan E. 2012. Alternative reading frame selection mediated by a tRNA-like domain of an internal ribosome entry site. Proc Natl Acad Sci USA 109:E630–9. doi:10.1073/pnas.1111303109 22247292 PMC3306683

[22] Wolfersberger MG. 1993. Preparation and partial characterization of amino acid transporting brush border membrane vesicles from the larval midgut of the gypsy moth (Lymantria dispar). Arch Insect Biochem Physiol 24:139–147. doi:10.1002/arch.940240304 7903055

[23] Bonning BC. 2025. Pathogen binding and entry: molecular interactions with the insect gut. Annu Rev Entomol 70:165–184. doi:10.1146/annurev-ento-030624-014608 39874144

[24] Mishra R, Guo Y, Kumar P, Cantón PE, Tavares CS, Banerjee R, Kuwar S, Bonning BC. 2021. Streamlined phage display library protocols for identification of insect gut binding peptides highlight peptide specificity. Curr Res Insect Sci 1:100012. doi:10.1016/j.cris.2021.100012 36003592 PMC9387513

[25] Ghosh AK, Ribolla PE, Jacobs-Lorena M. 2001. Targeting Plasmodium ligands on mosquito salivary glands and midgut with a phage display peptide library. Proc Natl Acad Sci USA 98:13278–13281. doi:10.1073/pnas.241491198 11687659 PMC60861

[26] Liu S, Sivakumar S, Sparks WO, Miller WA, Bonning BC. 2010. A peptide that binds the pea aphid gut impedes entry of Pea enation mosaic virus into the aphid hemocoel. Virology (Auckl) 401:107–116. doi:10.1016/j.virol.2010.02.009 20223498

[27] Sparks WO, Rohlfing A, Bonning BC. 2011. A peptide with similarity to baculovirus ODV-E66 binds the gut epithelium of Heliothis virescens and impedes infection with Autographa californica multiple nucleopolyhedrovirus. J Gen Virol 92:1051–1060. doi:10.1099/vir.0.028118-0 21228132

[28] Yang JY, Zhang Y. 2015. I-TASSER server: new development for protein structure and function predictions. Nucleic Acids Res 43:W174–W181. doi:10.1093/nar/gkv342 25883148 PMC4489253

[29] Kerr CH, Wang QS, Keatings K, Khong A, Allan D, Yip CK, Foster LJ, Jan E. 2015. The 5’ untranslated region of a novel infectious molecular clone of the dicistrovirus cricket paralysis virus modulates infection. J Virol 89:5919–5934. doi:10.1128/JVI.00463-15 25810541 PMC4442438

[30] Valles SM, Sabath N. 2012. No evidence for translation of pog, a predicted overlapping gene of Solenopsis invicta virus 1. Virus Genes 45:84–89. doi:10.1007/s11262-012-0746-5 22528643

[31] Chougule NP, Li H, Liu S, Linz LB, Narva KE, Meade T, Bonning BC. 2013. Retargeting of the Bacillus thuringiensis toxin Cyt2Aa against hemipteran insect pests. Proc Natl Acad Sci USA 110:8465–8470. doi:10.1073/pnas.1222144110 23650347 PMC3666667

[32] Vega-Rodríguez J, Ghosh AK, Kanzok SM, Dinglasan RR, Wang S, Bongio NJ, Kalume DE, Miura K, Long CA, Pandey A, Jacobs-Lorena M. 2014. Multiple pathways for Plasmodium ookinete invasion of the mosquito midgut. Proc Natl Acad Sci USA 111:E492–500. doi:10.1073/pnas.1315517111 24474798 PMC3910608

[33] Leonard SP, Powell JE, Perutka J, Geng P, Heckmann LC, Horak RD, Davies BW, Ellington AD, Barrick JE, Moran NA. 2020. Engineered symbionts activate honey bee immunity and limit pathogens. Science 367:573–576. doi:10.1126/science.aax9039 32001655 PMC7556694

[34] Altschul SF, Gish W, Miller W, Myers EW, Lipman DJ. 1990. Basic local alignment search tool. J Mol Biol 215:403–410. doi:10.1016/S0022-2836(05)80360-2 2231712

[35] Javed MA, Coutu C, Theilmann DA, Erlandson MA, Hegedus DD. 2019. Proteomics analysis of Trichoplusia ni midgut epithelial cell brush border membrane vesicles. Insect Sci 26:424–440. doi:10.1111/1744-7917.12547 29064633 PMC7379565

[36] Tavares CS, Mishra R, Ghobrial PN, Bonning BC. 2022. Composition and abundance of midgut surface proteins in the Asian citrus psyllid, Diaphorina citri. J Proteomics 261:104580. doi:10.1016/j.jprot.2022.104580 35427801

[37] Garczynski SF, Adang MJ. 1995. Bacillus thuringiensis CryIA(c) δ-endotoxin binding aminopeptidase in the Manduca sexta midgut has a glycosyl-phosphatidylinositol anchor. Insect Biochem Mol Biol 25:409–415. doi:10.1016/0965-1748(94)00072-7

[38] Carrillo-Tripp J, Dolezal AG, Goblirsch MJ, Miller WA, Toth AL, Bonning BC. 2016. In vivo and in vitro infection dynamics of honey bee viruses. Sci Rep 6:22265. doi:10.1038/srep22265 26923109 PMC4770293

[39] Lamp B, Url A, Seitz K, Eichhorn J, Riedel C, Sinn LJ, Indik S, Köglberger H, Rümenapf T. 2016. Construction and rescue of a molecular clone of deformed wing virus (DWV). PLoS ONE 11:e0164639. doi:10.1371/journal.pone.0164639 27828961 PMC5102418

[40] Schneider CA, Rasband WS, Eliceiri KW. 2012. NIH Image to ImageJ: 25 years of image analysis. Nat Methods 9:671–675. doi:10.1038/nmeth.2089 22930834 PMC5554542

