# Supplementary material for: Gut-binding peptides as potential tools to reduce virus binding to honey bee gut surface proteins
Source: Appl Environ Microbiol. 2025 Feb 28;91(3):e02418-24. doi: 10.1128/aem.02418-24 (PMC11921348; doi:10.1128/aem.02418-24)
Supplement: Supplemental material — Figures S1 to S5, Tables S1 to S4, and supplemental methods. [file aem.02418-24-s0001.pdf]

Gut binding peptides as potential tools to reduce virus binding to the honey bee gut epithelium

Supporting Information

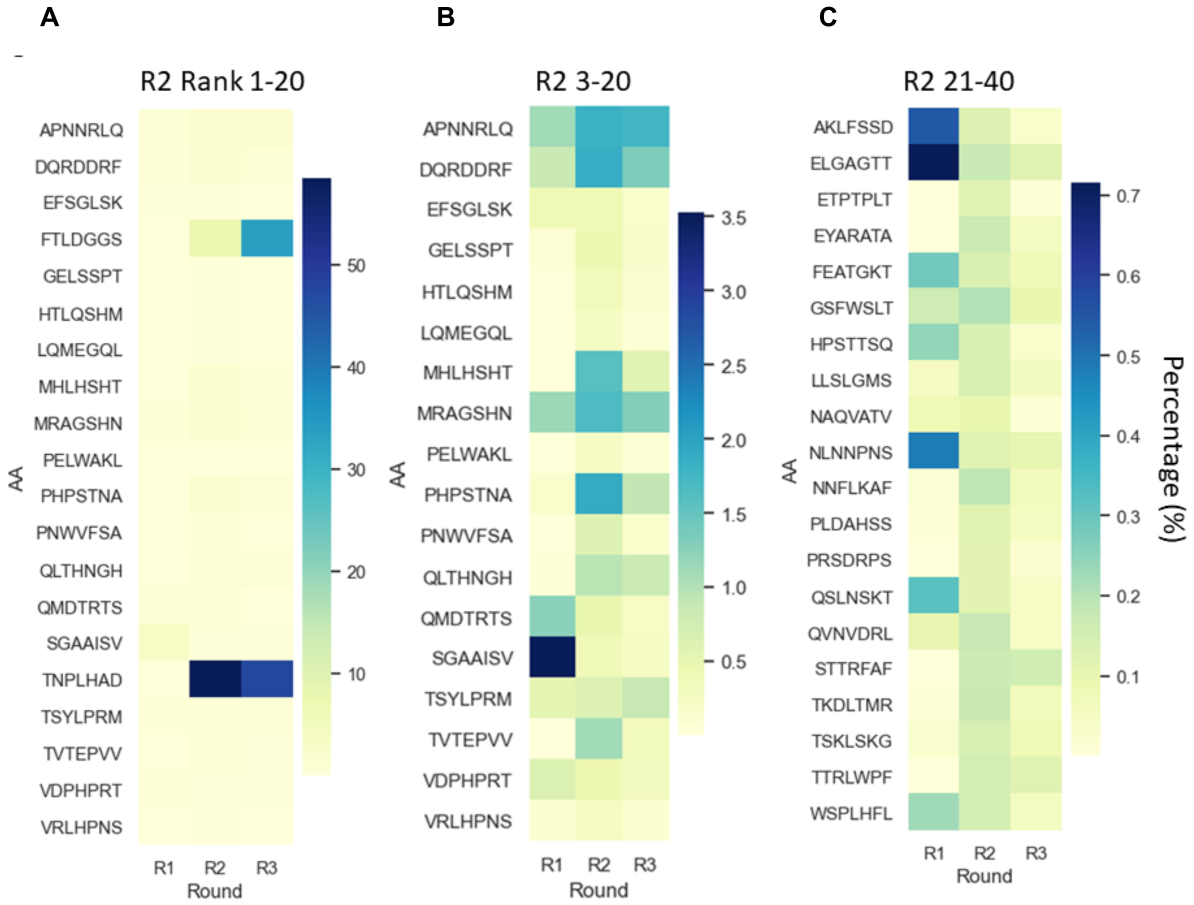

Figure S1. Changes in relative abundance of phage expressing the top 40 honey bee gut binding peptides during successive rounds of enrichment. This schematic highlights how the relative abundance of phage expressing the 40 most abundant peptides after round 2 of enrichment (R2) changed over successive rounds of phage selection, from R1 to R3 (x axes). A), the 20 most abundant peptides highlighting the two peptides with enrichment to >10% of all enriched peptides. Relative abundance at B) 3.5% or less for peptides with abundance ranked 3 to 20, and C) at 0.7% or less for peptides ranked 21 to 40. The peptide sequences for all 40 phage-displayed peptides are provided along with heatmap keys for percentage of all enriched peptides for each peptide group.

Table S1. **Summary data for the three most enriched peptides following each round of phage enrichment.**

	**1st round**			**2nd round**			**3rd round**			**Control[1]**		
	**Rank[2]**	**Count[3]**	**%**	**Rank**	**Count**	**%**	**Rank**	**Count**	**%**	**Rank**	**Count**	**%**
BBP1.1	1	157561	3.512	19	17476	0.357	15	9934	0.248	NA		
BBP1.2	2	70649	1.574	10	32037	0.654	10	23065	0.577	74	765	0.015
BBP1.3	3	64641	1.440	333	525	0.010	879	13	0.0003	NA		
**BBP2.1**	**10016**	12	**0.00026**	**1**	2848349	**58.18**	**1**	1901335	**47.56**	**496**	10	**0.000206**
**BBP2.2**	**12130**	8	**8.26E-05**	**2**	374500	**7.650**	**2**	1337199	**33.45**	**915**	4	**8.26E-05**
BBP2.3	127	6472	0.144	3	93101	1.901	6	36734	0.919	NA		
BBP3.3	7	49714	1.108	5	88976	1.817	3	69685	1.743	NA		
C1	33	22705	0.506	80	2969	0.060	151	601	0.015	1	2169329	44.78
C2	23832	2	4.46E-05	3604	4	8.17E-05	1967	2	5E-05	2	1327094	27.40
C3	36374	1	2.23E-05	1029	72	0.001	4912	1	2.5E-07	3	508763	10.50
**…**	**…**			**…**			**…**					
**Total**	**56032**	**4486263**		**13620**	**4895144**		**6053**	**3997038**		**4530**	**4843337**	

[1] Control, original naïve phage library; [2]Rank, relative abundance of peptide following round n of enrichment; [3]Count, number of times peptide sequence appears in sequence data following round n of enrichment.

Table S2. **Summary data for honey bee gut binding peptides with similarity to DWV or IAPV structural protein sequences**.

		PhD7Faster		PSBinder									
Peptide	AA sequence	Probability	Yes/No	Probability	Yes/No	Instability Index	GRAVY	R1 AA Count	R1 log2fold change	R2 AA Count	R2 log2fold change	R3 AA Count	R3 log2fold change
**BBP2.1**	TNPLHAD	0.95	Yes	0.13	**No**	**-29.14**	**-0.98**	12	1	2848349	18.10	1901335	17.81
**BBP1.15**	ELGAGTT	0.33	No	0.63	Yes	-15.68	-0.014	31954	14.96	8484	13.05	4732	12.50
**BBP1.3**	TPSGLFR	0.66	Yes	0.79	Yes	36.1	-0.2	64641	15.98	525	9.04	13	3.99
**BBP2.20**	HTLQSHM	0.33	No	0.09	No	107.14	-0.81	14	3.807	17204	14.07	4649	12.48
**BBP3.17**	TLPHSGW	0.98	Yes	0.52	Yes	53.71	-0.543	NA	1	7314	12.83	8170	13.29
**BBP2.2**	FTLDGGS	0.33	No	0.47	No	26.2	0.114	8	1.110	374500	16.49	1337199	18.62
**BBP2.13**	PNWVFSA	0.34	No	0.94	Yes	18.37	0.286	47	5.55	30564	14.90	5847	12.81
**BBP1.18**	FLSRPGV	0.98	Yes	0.72	Yes	63.6	0.5	29713	14.859	2422	11.24	1832	11.13
**BBP2.3**	PHPSTNA	0.99	Yes	0.89	Yes	10.5	1.37	6472	12.66	93101	16.51	36734	15.46

-

PSBinder: predicts polystyrene binding; PhD7Faster: predicts fast-growing clones.

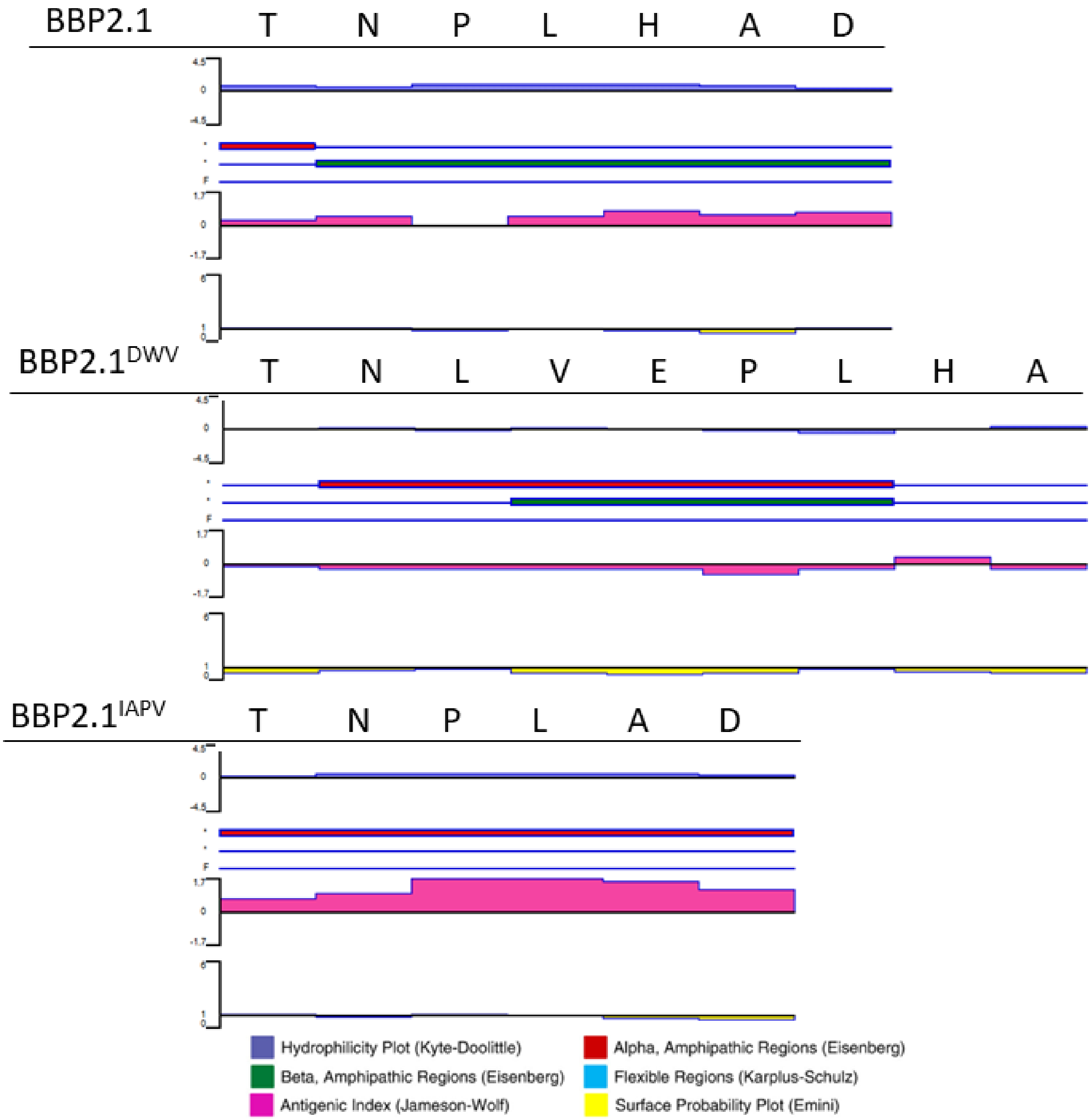

Figure S2. **Predicted properties of the three BBP2.1 peptides**. Structural features, hydrophilicity, antigenicity and surface probability profiles are shown for BBP2.1, BBP2.1^{DWV} and BBP2.1^{IAPV}. Generated using Protean prediction software (DNAstar Inc. v. 5.0). The antigenicity profiles were predicted using the Jameson–Wolf index. The y axes represent probability.

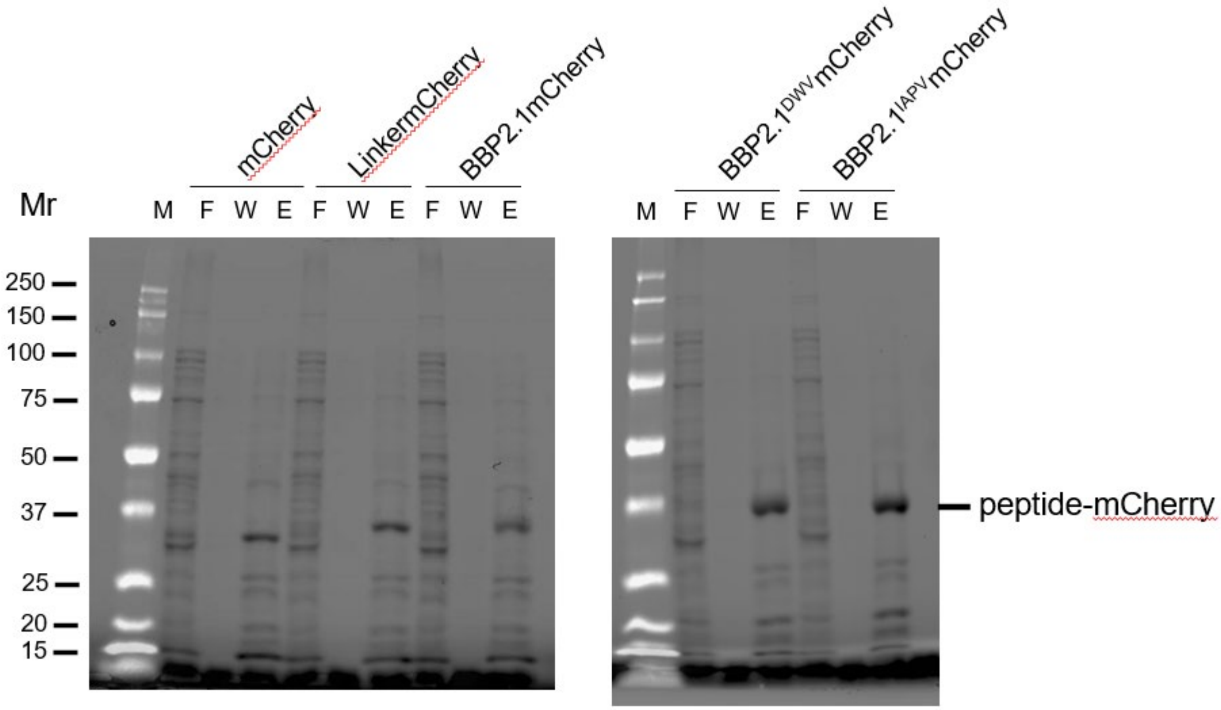

Figure S3. Purification of peptide-mCherry fusion proteins. SDS PAGE gel (10%) showing the flow through (F), wash (W) and eluted (E) proteins for peptide-linker-mCherry and control constructs used in pulldown assays with brush border membrane vesicles (BBMV; Figure 3). The Cooomassie Blue-stained gels provide an indication of relative purity of the protein samples and reflect the expected molecular mass for each protein; mCherry: 29.7 kDa; Linker-mCherry: 30.6 kDa; BBP2.1mCherry: 31.5 kDa; BBP2.1^{DWV}mCherry: 31.7 kDa; BBP2.1^{IAPV}mCherry: 31.4 kDa. Molecular mass markers (Mr) are provided (kDa).

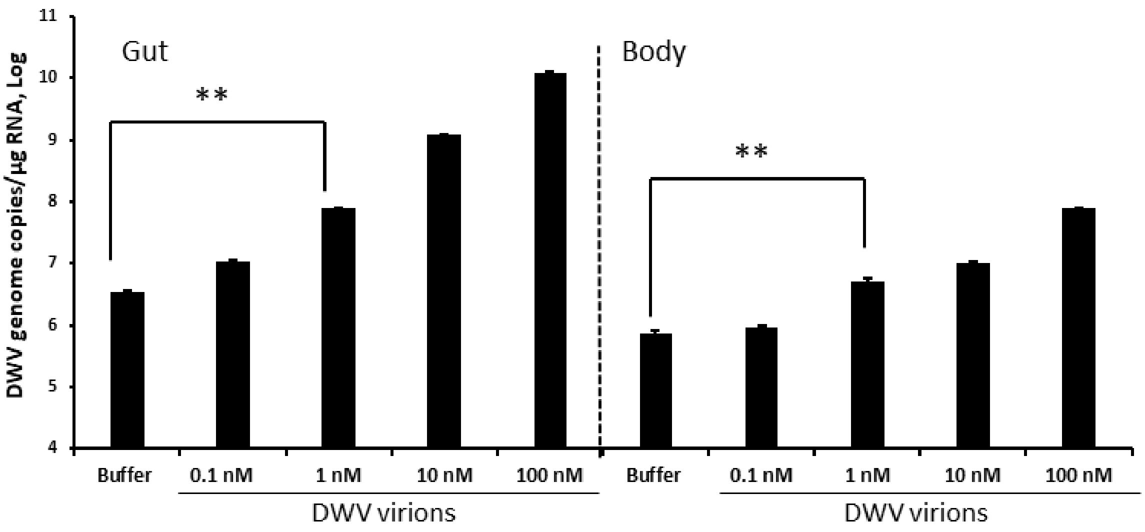

Figure S4. DWV inoculation dose required for detection of significantly increased viral titers above background DWV infection. Oral inoculation of honey bees with 1 nM DWV virions was sufficient to detect significantly increased viral loads against background DWV infection. Significant differences are indicated (Student's t-test).

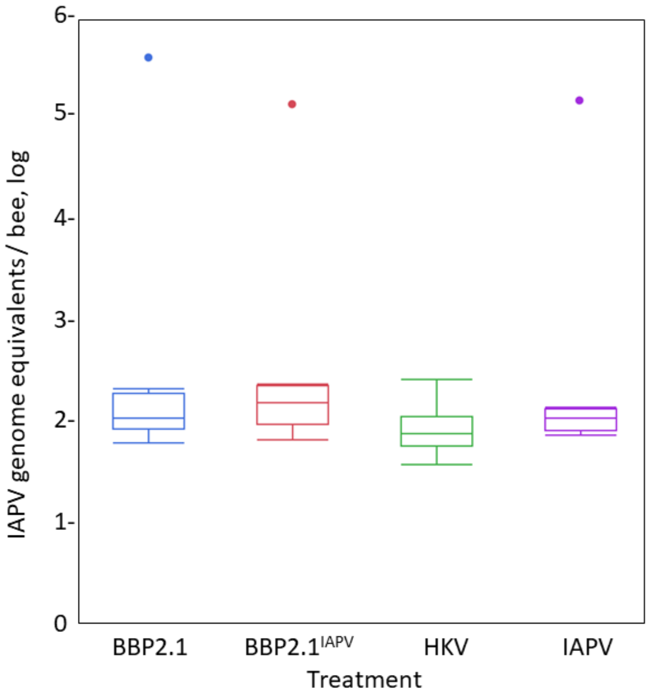

Figure S5. **BBP2.1 peptides did not reduce the IAPV load in treated honey bees.** No significant differences were noted in bees collected 36 hours post treatment between bees fed IAPV with or without BBP2.1 or BBP2.1^{IAPV}. The qPCR data are from a randomly selected subset of 8 bees from each treatment collected at 36 hpi (see Methods). Box plot bars show the upper and lower limits of the dataset, with the exception of outliers, indicated by dots. HKV, heat-killed virus.

Supplementary Methods

In vivo honey bee gut binding peptide isolation

A disulfide-constrained heptapeptide library (Ph.D.-C7C, New England Biolabs, Ipswich, MA, USA) was used to isolate honey bee gut binding peptides *in vivo* as described previously [1]. Additional details for the honey bee protocol are provided here.

A total of 15 newly emerged bees were collected. Individual bees were fed on the phage display library by droplet feeding of 20 μL TBS (pH 7.5) buffer containing 10^{11} pfu phage/mL and 30% sucrose. Treated bees were incubated at 32°C for 2 h, then midguts dissected. Midguts were suspended in 1 mL TBS (pH 7.5) containing 1% BSA, gently ground with a pestle in a microcentrifuge tube and centrifuged at 1500 g for 5 minutes at 4°C. The supernatant was removed, and the pellet resuspended in 1 mL TBST (TBS supplemented by 0.5% Tween-20) to remove unbound phages. These suspensions were centrifuged again and washed three times. Bound phages were eluted by adding 500 μL of elution buffer (50 mM glycine-HCl, pH 2.2, 1 mg/mL BSA) and rotating gently for 10-15 minutes at room temperature, followed by centrifugation at 1500×g for 5 min. The supernatant was transferred to a 1.5-mL tube and neutralized with the addition of 20 μL of 1 M Tris-HCl Buffer (pH 9.1). A small amount (~1 μL) of the eluate was titrated to estimate the number of recovered phages from each round of biopanning. The remaining eluted phages were amplified immediately in ER2738 cells according to the Ph.D.™ Phage Display Library instructions (New England Biolabs). The amplified phages were titrated and used for the next round of biopanning. Three rounds of phage enrichment were conducted.

Peptide sequences encoded by phages eluted after each round of enrichment were obtained using next generation sequencing (MiSeq; Genewiz, South Plainfield, NJ). Single-stranded DNA (ssDNA) was extracted from eluted phages according to the EZNA M13 mini kit protocol (Omega Bio-Tek, Norcross, GA, USA). A naïve phage display library from the same lot number was used as a negative control. Sequencing and bioinformatics details are provided by Mishra et al., 2021 [1].

1. Mishra, R., et al., *Streamlined phage display library protocols for identification of insect gut binding peptides highlight peptide specificity.* Current Research in Insect Science, 2021. **1**: p. 100012.

Table S3. **Primers used for production of peptide–mCherry fusions**. Underlined sequences: *Sac*I site in forward (F) primers, *Hind*III site in reverse (R) primer.

Primer	Sequence (5' to 3')
BBP2.1_mChSgF	AATGAGCTCGtgtacgaatccgcttcatgcggattgcgcaccagcccctgcaccagcccctgcaccaatggtgagcaagggcgagg
BBP2.1DWV_mChSgF	AATGAGCTCGtgtactaatttagttgaaccattacatgcatgcgcaccagcccctgcaccagcccctgcaccaatggtgagcaagggcgag
BBP2.1IAPV_mChSgF	AATGAGCTCGtgtacaaacccccttagccgattgcgcaccagcccctgcaccagcccctgcaccaatggtgagcaagggcgagg
LinkermChSgF	AATGAGCTCGgcaccagcccctgcaccagcccctgcaccaatggtgagcaagggcgagg
mChcHR	ATTAAGCTTCtacttgtacagctcgtccatgcc
mChSgF	ATTGAGCTCGatggtgagcaagggcgagg

Table S4. Primers used for the detection of honey bee viruses[1].

Viruses	Primers	Sequences (5’-3’)
Deformed wing virus	DWVqRTPCR-F	GAGATTGAAGCGCATGAACA
	DWVqRTPCR-R	TGAATTCAGTGTCGCCCATA
Israeli acute paralysis virus	IAPVqRTPCR-2F	GCACAGTCTTCTGGTGATTGC
	IAPVqRTPCR-2R	GTTAGCACACGATTGGTTATCAGC
Acute bee paralysis virus	ABPVqRTPCR-F	ACCGACAAAGGGTATGATGC
	ABPVqRTPCR-R	CTTGAGTTTGCGGTGTTCCT
Black queen cell virus	BQCVqRTPCR-F	TTTAGAGCGAATTCGGAAACA
	BQCVqRTPCR-R	GGCGTACCGATAAAGATGGA
Kashmir bee virus	KBVqRTPCR-F	TGGCATCGAGCGCATTCCAG
	KBVqRTPCR-R	TCGGGGTTTGGACCACCCGAAT
Sacbrood bee virus	SBVqRTPCR-F	GGGTCGAGTGGTACTGGAAA
	SBVqRTPCR-R	ACACAACACTCGTGGGTGAC

[1] From: Carrillo-Tripp, J., et al., *In vivo and in vitro infection dynamics of honey bee viruses.* Sci Rep, 2016. **6**: p. 22265.
